# Modern Sociology

**Published:** 1899-10-07

**Authors:** 


### Modern Sociology.

### COMPULSORY SCHOOLING OR COMPULSORY EDUCATION?

Two of the boasts of the Victorian age are that child-labour has been abolished and compulsory education introduced. But when we read Sir John Gorst's statement on child labour, as laid before the House of Commons, we begin to wonder if the boast is justified. A child of school age may not lawfully be kept from school to work for wages. But what control has the teacher, or the School Board, or the Education Department itself, over those who go to school and work out of school hours. Yet this is what many children do. They are worse off than even the half-timers, for these are employed only half the day at work and half at school; but the children we speak of have an entire school day, and yet work from dawn to school time, and from the dismissal of the school till midnight. It is impossible to say how many children are thus employed. The schedules which the Education Department sent out with questions to be answered on this subject were most imperfectly filled up. The question was, How many children in each district were working for profit? But many school managers translated "profit" into "wages," and gave no information as to children who were helping their parents in doing paid work, though these are probably as numerous as those who are working for wages paid by strangers. Among this class there is also much irregularity of attendance; but this, though unfavourable to education, is probably less prejudicial to health than the combination of school lessons and home work.

In the country districts children are almost invariably kept away from school during harvest. Wise school managers, with an eye to the effect of average attendance on grants, arrange to give the annual summer holidays at that time; but they cannot reckon with all the exigencies of weeding, bird-scaring, potato-picking, and the like. In seaside resorts the summer days are otherwise employed. The parents cater for excursionists, and the children are occupied in touting for orders, and assisting in the sale of refreshments, or in looking after the dilapidated donkeys on which the excursionist enjoys (?) a penny ride. "The result is," says one school manager, "that at least 70 per cent. of our children are in some way employed out of school hours between June and October." Besides these, there are always children employed running errands, selling newspapers, and—alas that a healthy amusement should have so evil a result—as caddies on golf-links.

So much for boys. With regard to girls, it is needless to say that almost all have to help at home to some extent. There is always a baby to "mind;" if not their own, then that of some over-burdened neighbour, who may—or may not—pay the little nurse for her assistance. There is no use in discussing such tasks, for so long as woman's nature is what Providence meant it to be, so long will there be children willing to spend their leisure in such work. But in trades followed at home by women the girls of the family almost invariably take a share. The reports vary according to the industry of the district. "During the hat-sewing season, which usually lasts from February to Whitsuntide, many girls of all ages are employed, both before and after school hours, in sewing hats for their mothers. Some have been known to work from six a.m. to the time for coming to school, and again from school-closing till bedtime." "All the girls in the school, excepting a few, are employed out of school hours in assisting at glove-making. Earnings often considerable." "Many of the elder girls leave school when the summer season begins, and are employed in the lodging-houses, &c. They receive about 2s. 6d. per week," and so on.

In some cases the wages which the child receives are sufficient to account for the average parent of the labouring class thinking that they are worth more than education. Thus in Cornwall, there are boys who help the fishermen, and thereby earn about 10s. a week. But such earnings are altogether exceptional; and more generally one wonders why any child will work, or any parent allow a child to work, for such inadequate remuneration as they receive. Take the case of a girl who works in a shop for 72 hours a week for 2s. Another, a little girl under six years of age, is a nursemaid (poor nurse and poor nurseling), who earns the magnificent wage of twopence a week, in addition to her food, for 29 hours' work. Wages of less than sixpence a week are quite common, especially in London, though the industrial districts are almost as bad. And, of course, this return leaves untouched the numerous cases where no money wage is given, as where the children are helping in their parents' work, and their share of the family income is not separately reckoned.

Naturally, when they do come to school, they are not fit to learn much. Many, who have been up and at work for three or four hours before school begins, fall asleep in school; and even when their eyes keep open it cannot be expected that their minds are awake. No wonder people ask what good education does such children. Clearly it does very little. But that is not the fault of the education; nor could we reconcile it with the national conscience to give up the attempt to educate the children of our country because they or their parents do not take full advantage of the teaching given. That would be merely to give up the problem, not to solve it.

How then can it be solved? In Liverpool an attempt has been made. There, in future, no child under the age of eleven is to be allowed to sell things in the streets, and children over eleven must obtain a licence, without which they will not be allowed to sell. But this, at best, only touches one part of the evil; and it is difficult to see how outside influences can deal with it. The compulsory officer can deal with truants, and parents are punished who do not send their children to school. It is true that sharper treatment, especially a larger fine, is sometimes needed in order to keep parents up to the mark. If they can pay a fine out of the child's earnings and have even a little over they are content. It is essential that the fine should swallow up all that the child can earn. But even if they can be thus compelled to send them to school, they may make them work so hard out of school hours that their education does little good to the weary mind in the fatigued body. Verily, what we want is something that will secure the compulsory moral education of parents, and teach them their duty to their offspring!

